# Multi-Design Differential Expression Profiling of COVID-19 Lung Autopsy Specimens Reveals Significantly Deregulated Inflammatory Pathways and SFTPC Impaired Transcription

**DOI:** 10.3390/cells11061011

**Published:** 2022-03-16

**Authors:** Matteo Fassan, Antonio Collesei, Valentina Angerilli, Marta Sbaraglia, Francesco Fortarezza, Federica Pezzuto, Monica De Gaspari, Gianluca Businello, Margherita Moni, Stefania Rizzo, Giulia Traverso, Veronica Colosso, Elisa Taschin, Francesca Lunardi, Aida Freire Valls, Francesca Schiavi, Cristina Basso, Fiorella Calabrese, Angelo Paolo Dei Tos

**Affiliations:** 1 Department of Medicine (DIMED), University of Padua, 35121 Padua, Italy; valentina.angerilli@gmail.com (V.A.); marta.sbaraglia@aopd.veneto.it (M.S.); glc.businello@gmail.com (G.B.); margherita.moni@studenti.unipd.it (M.M.); me.giulia@gmail.com (G.T.); francesca.lunardi@unipd.it (F.L.); angelo.deitos@unipd.it (A.P.D.T.); 2 Surgical Pathology Unit, Padua University Hospital, 35121 Padua, Italy; francesco.fortarezza@unipd.it (F.F.); federica.pezzuto@unipd.it (F.P.); fiorella.calabrese@unipd.it (F.C.); 3 Veneto Institute of Oncology, IOV-IRCCS, 35128 Padua, Italy; 4 Familial Cancer Clinics, Veneto Institute of Oncology, IOV-IRCCS, 35127 Padua, Italy; antonio.collesei@iov.veneto.it (A.C.); veronica.colosso@iov.veneto.it (V.C.); elisa.taschin@iov.veneto.it (E.T.); francesca.schiavi@iov.veneto.it (F.S.); 5 Department of Surgery, Oncology and Gastroenterology, University of Padua, 35121 Padua, Italy; 6 Department of Cardiac, Thoracic, Vascular Sciences and Public Health, University of Padua, 35121 Padua, Italy; monica.deg1@gmail.com (M.D.G.); s.rizzo@unipd.it (S.R.); cristina.basso@unipd.it (C.B.); 7 Cardiovascular Pathology Unit, Padua University Hospital, 35121 Padua, Italy; 8 NanoString Technologies, Inc., 530 Fairview Avenue N, Seattle, WA 98109, USA; avalls@nanostring.com

**Keywords:** SARS-CoV-2, COVID-19, autopsy, transcriptomic profiling, inflammation, complement

## Abstract

The transcriptomic profiling of lung damage associated with SARS-CoV-2 infection may lead to the development of effective therapies to prevent COVID-19-related deaths. We selected a series of 21 autoptic lung samples, 14 of which had positive nasopharyngeal swabs for SARS-CoV-2 and a clinical diagnosis of COVID-19-related death; their pulmonary viral load was quantified with a specific probe for SARS-CoV-2. The remaining seven cases had no documented respiratory disease and were used as controls. RNA from formalin-fixed paraffin-embedded (FFPE) tissue samples was extracted to perform gene expression profiling by means of targeted (Nanostring) and comprehensive RNA-Seq. Two differential expression designs were carried out leading to relevant results in terms of deregulation. SARS-CoV-2 positive specimens presented a significant overexpression in genes of the type I interferon signaling pathway (IFIT1, OAS1, ISG15 and RSAD2), complement activation (C2 and CFB), macrophage polarization (PKM, SIGLEC1, CD163 and MS4A4A) and Cathepsin C (CTSC). CD163, Siglec-1 and Cathepsin C overexpression was validated by immunohistochemistry. SFTPC, the encoding gene for pulmonary-associated surfactant protein C, emerged as a key identifier of COVID-19 patients with high viral load. This study successfully recognized SARS-CoV-2 specific immune signatures in lung samples and highlighted new potential therapeutic targets. A better understanding of the immunopathogenic mechanisms of SARS-CoV-2 induced lung damage is required to develop effective individualized pharmacological strategies.

## 1. Introduction

Severe acute respiratory syndrome coronavirus-2 (SARS-CoV-2) is responsible for coronavirus disease 2019 (COVID-19) pandemic: as of November 2021, over 200 million people have been infected and there have been over 5 million deaths.

It is well-established that COVID-19 progression is driven by an inflammatory syndrome caused by an hyperactivation of the immune system. Therefore, disease severity in patients is due to not only the viral infection, but also to the variability of host innate and adaptive immune response, which can result in a wide spectrum of clinical outcomes, ranging from absence of symptoms to severe disease, multiple organ failure, and ultimately death [1,2].

A combination of cytopathic effect, persistent inflammation and immune system activation is responsible for lung tissue damage, resulting in dyspnea, acute lung injury (ALI) and respiratory distress syndrome (ARDS) [3,4].

Understanding COVID-19 related-pathology and the molecular and cellular mechanisms underlying the physiopathology of the disease is essential to obtain reliable diagnostics and optimize clinical management. Because it is rare to obtain samples from living patients, autoptic specimens are essential to gain knowledge of severe COVID-19 lung pathology. Many nonspecific histopathological findings have been identified in autoptic specimens, such as diffuse alveolar damage (DAD), lymphomonocytic and neutrophilic infiltrate, and pleural effusion [5,6].

In this work, we used histologic, immunohistochemical and transcriptomic analyses of post mortem lung tissues, to gain insight into the immunopathologic landscape of COVID-19 lung disease and putative therapeutic strategies targeting aberrant immune responses.

## 2. Materials and Methods

### 2.1. Patient Selection

Lung tissue samples were obtained from 24 patients (17 cases and 7 controls) who died between 20 March 2020 and 30 May 2020 at Padua University Hospital.

The cases were patients with a SARS-CoV-2 infection confirmed by real-time PCR analysis of nasopharyngeal swab samples taken at the time of hospital admission and who died from COVID-19. The controls were deceased patients without any clinically documented respiratory disease.

### 2.2. Autopsy and Tissue Processing

As a safety precaution against infection, a COVID-19-optimized autopsy protocol was followed, in line with the recently published recommendations of the Italian Health Department [7,8].

A median of 5 (range 3 to 7) lung tissue blocks were sampled. All tissue samples were processed according to standard protocols, with formalin fixation time > 48 h. Paraffin-embedded sections of 3 μm thickness were stained with hematoxylin and eosin (H&E). Histological examination of COVID-19 pneumonia was performed taking into account several morphological features, as previously described [9,10].

Three experienced pathologists (MF, FP and FC) jointly evaluated the H&E slides to select the most representative tissue block to perform gene-expression profiling and immunohistochemistry (IHC).

### 2.3. RNA Qualification

Five consecutive 10-μm thick sections from each selected FFPE tissue block were obtained. The RecoverAll™ Total Nucleic Acid Isolation Kit (Thermo Fisher Scientific, Waltham, MA, USA) was used to isolate RNA from the material, according to the manufacturer’s instructions. RNA concentration was determined using Qubit^®^ 2.0 Fluorometer (Life Technology, Carlsbad, CA, USA). RNA quality was assessed on the Agilent Bioanalyzed 2100 (Agilent Technologies, Santa Clara, CA, USA), according to the DV200 parameter.

### 2.4. Nanostring Gene-Expression Profiling

At least 100 ng of total RNA was loaded for hybridization with the nCounter^®^ Autoimmune Profiling Panel and COVID Plus Panel (NanoString Technologies, Seattle, WA, USA) for 16 h at 65 °C, according to the manufacturer’s instructions and quantified by the nCounter^®^ MAX Analysis System (NanoString Technologies). Raw data processing, quality control, and normalization were performed using the nSolver™ 4.0 Analysis Software (NanoString Technologies). Transcript counts were normalized to housekeeper reference gene expression prior to analysis. Three samples out of the 17 SARS-CoV-2 cases were excluded from the analysis due to low quality of the run.

### 2.5. RNA-Seq Gene-Expression Profiling

Total RNA was profiled using SMARTer^®^ Stranded Total RNA-Seq kit v2- Pico Input Mammalian (Takara Bio USA, Inc., San Jose, CA, USA). Briefly, 50 ng of total RNA was reverse transcribed to single strand cDNA using random primers. Illumina adapters and indexes were added through PCR using only a limited number of cycles. The PCR products were purified using AMPure Beads and then the ribosomal cDNA was depleted. The resulting ribo-depleted library fragments were enriched in a second round of PCR. The amplified RNA-Seq library was purified by immobilization onto AMPure beads and was eluted in TRIS buffer. Purified RNA-Seq library was quantified with Qubit dsDNA HS kit (Thermo Fisher Scientific), run on an Agilent 4200 TapeStation D1000 HS ScreenTape (Agilent, Santa Clara, CA, USA) to assess size distribution of the library molecules, and pooled to equimolar concentration. Pooled RNA-Seq libraries were sequenced with paired-end 75-bp reads using an Illumina NextSeq 550 sequencer (Illumina, Inc., San Diego, CA, USA) and raw data were stored in BaseSpace Sequence Hub (Illumina).

### 2.6. Bioinformatic Analysis

Raw FASTQ data were mapped using the STAR aligner and gene counts were quantified with Salmon. Viral load and clade were generated through the Illumina DRAGEN COVID Lineage v 3.5.0 pipeline, included in the BaseSpace App suite, and served as metadata for the subsequent differential analysis.

Clades were assigned according to the nomenclature defined by Nextstrain.

Raw counts and metadata for each sample of interest were imported into the R environment (R version 4.0.5—Shake and Throw).

Genes with poor count levels were filtered out, keeping the ones with a mean value of at least 10, across all samples. Differential expression analysis was performed using the R package DESeq2 exploiting Wald test for statistical significance and Benjamini–Hochberg method for multiple testing correction.

Two design patterns were considered when performing differential expression analysis. Firstly, COVID-infected gene expression profiles were compared to pure control samples’ counts, related to deceased patients without any respiratory disease (virus-vs.-control design). Secondly, the two COVID subgroups, low and high viral load, were tested one against the other (low-vs.-high design).

### 2.7. Immunohistochemistry

Immunohistochemical (IHC) stains were performed using the Bond Polymer Refine Detection kit (Leica Biosystems, Newcastle upon Tyne, UK) in the BOND-MAX system (Leica Biosystems) with appropriate staining protocol. Four-μm-thick FFPE sections were incubated with the primary antibodies for CD163 (10D6, Leica Biosystems; prediluted), Siglec-1 (D-6, Santa Cruz Biotech; 1:400) and Cathepsin C (B-1, Santa Cruz Biotech; 1:100). The evaluation of the IHC-stained slides was performed by two expert pathologists (M.F. and MS).

The expression of CD163 and Siglec-1 was quantified by counting the positive macrophages in five consecutive 40× fields, sampling representative different regions of the sample. The mean value per high power field (HPF) for each sample was calculated by dividing the total number of positive cells by 5. When evaluating the expression of Cathepsin C, a semiquantitative scoring system was used as follows: 0 = absence of staining; 1 = weak staining; 2 = moderate staining and 3 = strong staining.

The differential expression of CD163, Siglec-1 and Cathepsin C between cases and controls was detected by applying the Wilcoxon–Mann–Whitney test. A *p*-value < 0.05 was considered significant. The statistical analysis was performed using the STATA software (Stata Corporation, College Station, TX, USA).

## 3. Results

### 3.1. Autoptic Evaluation of Lung from COVID-19 and Control Patients

We analyzed 24 post mortem lung samples from 17 deceased COVID-19 patients and post mortem lung samples from seven patients who died of cardiovascular diseases and had no clinically documented respiratory disease, nor infectious disease. All COVID-19 patients were confirmed for SARS-CoV-2 infection through qRT-PCR assays performed on nasopharyngeal swab specimens. The primary cause of death in all patients of the COVID-19 cohort was respiratory failure. Clinical summaries of the 24 patients are listed in Table 1.

Lungs from COVID-19 patients, macroscopically, were increased in volume, with bilateral interstitial oedema and congestion. The cut surfaces revealed tan-gray consolidations and patchy hemorrhagic areas. Microscopically, lungs showed nonspecific lesions consistent with a diagnosis of DAD: multifocal damage with both exudative and proliferative inflammation, inclusive of hyaline membrane formation, alveolar-capillary barrier injury with red blood cell extravasation, inflammatory cells infiltration into the intra-alveolar space, fibroblast and myofibroblast proliferation, acute fibrinous organizing pneumonia and organizing pneumonia, extracellular matrix deposition, parenchymal remodeling, and pulmonary fibrosis. Pneumocytes squamous metaplasia and microthrombi in capillary beds and arterioles were reported in 3 and 11 cases respectively. Lungs from controls showed, apart from congestion and focal oedema, no other relevant pathological aspects.

Regarding the clinical/laboratory information of COVID-19 patients, main characteristics are summarized in Table 2. No significant clusterization of the samples was obtained by considering clinical data (i.e., timing of intubation, type of therapy), sex or age (75 y and 80 y as cut-offs).

### 3.2. Detection of SARS-CoV-2 and Clades Identification

Based on the quantitative assessment of SARS-CoV-2-related genes’ expression, six cases were classified with a high viral load. There was concordance between RNA-Seq and digital multiplexed gene expression technologies for determining samples with high viral load.

The sequencing approach also enabled insights into the genetics of the high viral load SARS-CoV-2 samples, classified into a total of 3 clades 19A (patients S08, S10), 20A (S17) and 20B (S01, S14, S15) (Supplementary Figure S1).

### 3.3. Gene Expression Profiling Identified an Hyperinflammatory Status of the Lung Parenchyma and a Dysregulation in the Complement Signaling

One representative lung specimen for each patient was subject to gene expression profiling by using a commercially available nCounter panel (nCounter^®^ Autoimmune Profiling Panel and COVID Plus Panel), to investigate 752 (plus 20 reference genes) differentially expressed immune and inflammatory-related transcripts. The transcriptomic signature identified by the nCounter analysis was further validated by total RNA-Seq, which is able to test for most of 30,000 transcripts.

Three samples of the COVID-19 cohort were discarded from the analysis due to poor quality of RNA (RNA integrity number—RIN < 3) [11].

**Figure 1:**
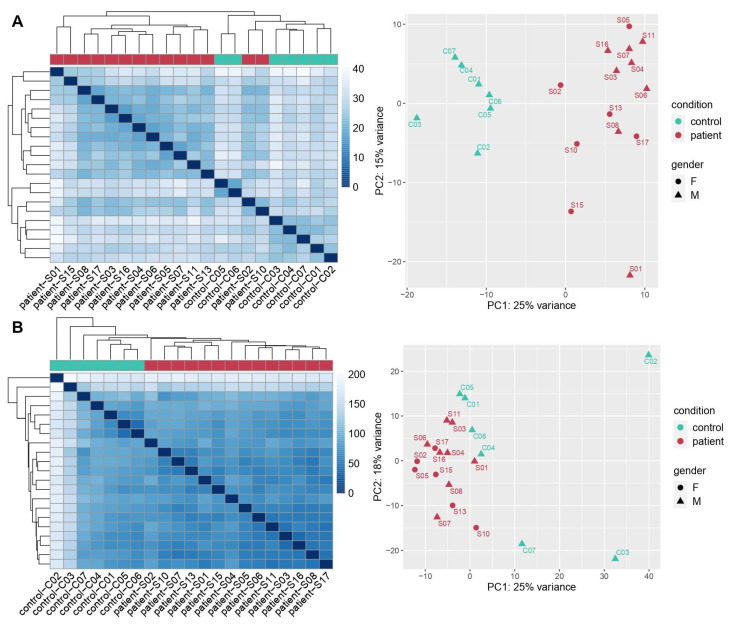
Cases distribution according to distance-based hierarchical clustering heatmaps and principal component analysis (PCA) graphs related to Nanostring (**A**) and total RNA-Seq (**B**) results, respectively. The heatmaps are a color-coding graphical representation of data coming from transcriptome analyses according to differentially expressed genes (DEGs) distribution. These plots were built using the Pheatmap package in R achieved by hierarchical, agglomerative clustering methods. The PCA graph depicts variation within and between the two groups. Both representations for the two methods of analysis showed a clear separation between COVID-19 patients (samples) and controls.

Both Nanostring and RNA-Seq analysis were able to discriminate between COVID-19 patients and control samples (Figure 1). The hierarchical clustering heatmaps and the principal component analysis (PCA) graphs based on the differentially expressed genes (DEGs) depict a clear separation of the two groups with no significant difference between male and females.

**Figure 2:**
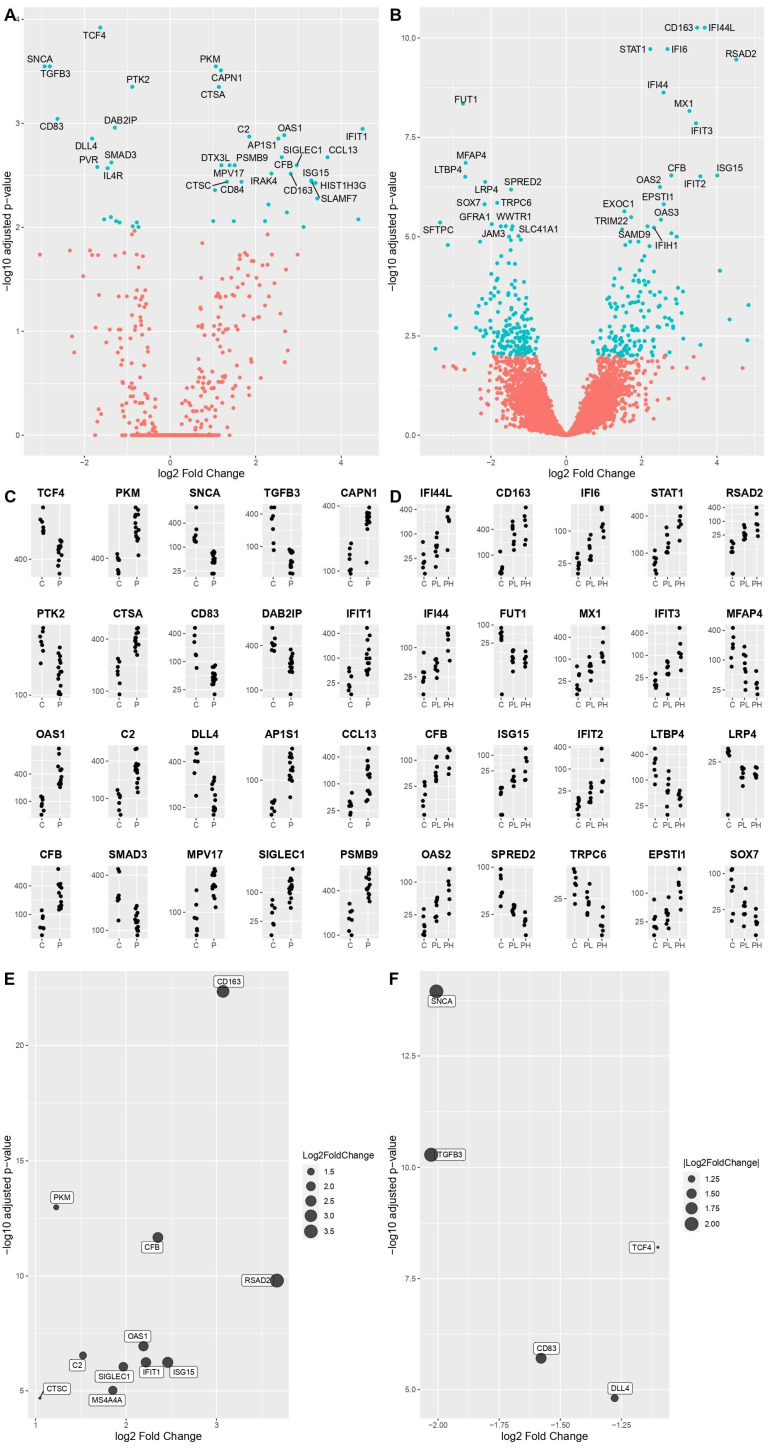
The volcano plots depict the DEGs in virus-vs.-control design according to nCounter (**A**) and RNA-Seq (**B**) analyses. Specifically, on the right part of the plot COVID-19 significantly overexpressed genes are highlighted in terms of statistical relevance, on the left the downregulated ones. Extreme positions on the *x*-axis mean higher log2 fold change. Panels (**C**,**D**) are showing the relative expression of the top 20 most deregulated genes, in the same design, in the nCounter and RNA-Seq methodologies, respectively (C = control, P = patient with virus in low and high load). Extended analysis of the commonly deregulated genes in the two methodologies is shown in panel (**E**) (most expressed) and (**F**) (least expressed), with the largest dots representing increasingly higher values of log2 fold change.

DEGs were identified by comparing COVID-19 patients’ transcriptomes with the controls’ transcriptomes. An adjusted *p*-value (*q*-value < 0.05) and fold change (FC) ratio (|log2FC| ≥ 2) were used to determine the DEGs. The volcano plots (Figure 2A,B) highlight the DEGs in virus versus control design both in nCounter and RNA-Seq experiment, and the specific genes with the highest degree of statistical significance in terms of differential expression. The relative expression of the top 20 most deregulated genes for nCounter analysis is shown in Figure 2C, while in Figure 2D the results of virus versus control RNA-Seq experiment are depicted differentiating between controls, and samples with low and high viral load.

According to nCounter analysis, the genes with higher expression levels amongst the samples were: *PKM*, *CAPN1*, *CTSA*, *IFIT1*, *OAS1*, *C2*, *AP1S1*, *CCL13*, *CFB*, *MPV17*, *SIGLEC1* and *PSMB9*. On the other hand, differential expression analysis revealed a lower level of expression in the following genes: *TCF4*, *SNCA*, *TGFB3*, *PTK2*, *CD83*, *DAB2IP*, *DLL4* and *SMAD3*. For the purpose of our study, we divided these DEGs into macrocategories of pathways: INF I signaling pathway (to which *IFIT1*, *OAS1*, *CCL13* and *DAB2IP* belong), macrophage activation genes (to which *PKM*, *SIGLEC1*, *PSMB9* and *CD83* belong), complement system (to which *C2* and *CFB* belong), Cathepsin genes (*CTSA*) and other genes (*CAPN1*, *AP1S1*, *MPV17*, *TNF4*, *SNCA*, *TGFB3*, *PTK2*, *DLL4*, *SMAD3*).

According to RNA-Seq data, the genes with a significantly higher expression level in COVID-19 related samples, were *IFI44L*, *CD163*, *IFI6*, *STAT1*, *RSAD2*, *IFI44*, *MX1*, *IFIT3*, *CFB*, *ISG15*, *IFIT2*, *OAS2* and *EPSTI1*. On the other hand, differential expression analysis revealed lower levels in: *FUT1*, *MFAP4*, *LTBP4*, *LRP4*, *SPRED2*, *TRPC6* and *SOX7*. According to the aforementioned pathways’ categories, these genes can be divided into: INF I signaling pathway (to which *IFI44L*, *IFI44*, *IFI6*, *STAT1*, *RSAD2*, *MX1*, *IFIT3*, *ISG15*, *IFIT2* and *OAS2* belong), macrophage activation genes (to which CD163 and EPSTI1 belong), complement system (*CFB*) and other genes not included in the categories listed above (*FUT1*, *MFAP4*, *LTBP4*, *LRP4*, *SPRED2*, *TRPC6* and *SOX7*).

We then expanded the analysis to all significantly deregulated genes in both methodologies. We found an important overlap, confirming the relevance of the following genes. The commonly overexpressed ones were: *PKM*, *IFIT1*, *OAS1*, *C2*, *CFB*, *SIGLEC1*, *CD163*, *ISG15*, *CTSC*, *RSAD2* and *MS4A4A*, while the commonly downregulated ones: *TCF4*, *SNCA*, *TGFB3*, *CD83* and *DDL4* (Figure 2E,F). Table 3 lists the common increased and decreased DEGs from both the nCounter and total RNA-Seq analyses.

### 3.4. Major Cell Type Components of SARS-CoV-2 Associated Inflammation

**Figure 3:**
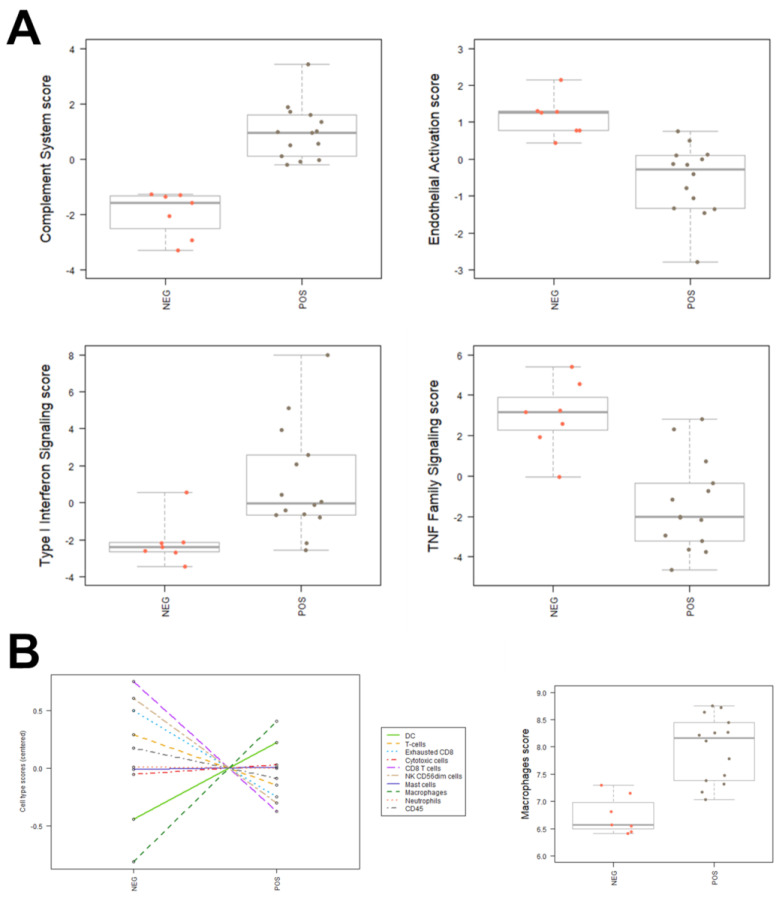
Main inflammatory pathways and cell types involved in SARS-CoV-2 infection. (**A**) Boxplots of effectors of complement system, endothelial activation, type I interferon signaling, TNF family signaling and macrophages in samples and controls. (**B**) Distribution of cell types between samples (pos) and controls (neg).

The nCounter analysis allows to discriminate the main pathways and main cell types involved in the inflammatory insult. In particular, the COVID-19-related samples were characterized by a significant activation of genes related to type I interferon signaling pathway, the complement system, and macrophages (Figure 3A). Endothelial activation genes and TNF family signaling pathways are downregulated in the samples in comparison to controls. The distribution of various cell types between samples and controls, obtained by processing Nanostring raw data, showed a more abundant population of DCs and macrophages in the COVID-19 related samples (Figure 3B).

### 3.5. Comparison between Lung Samples with Different Viral Load (Low versus High Design)

**Figure 4:**
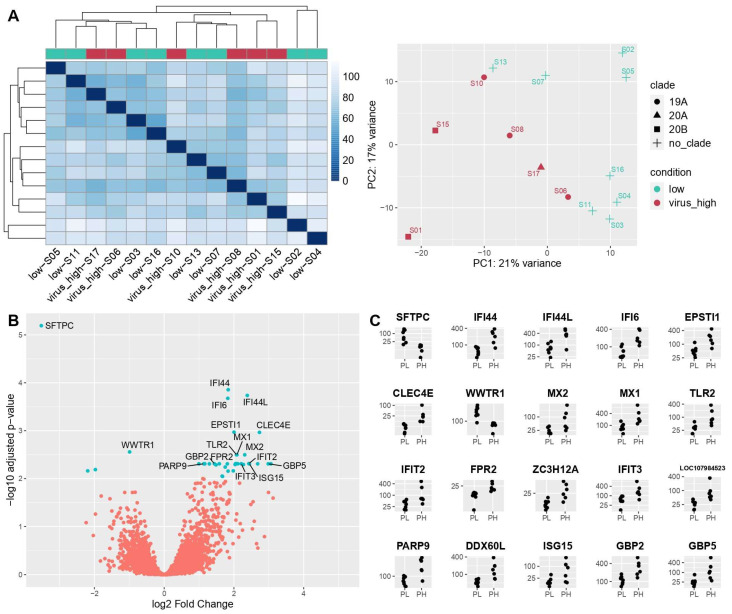
(**A**) Distance-based heatmap and PCA graph of RNA-Seq results in low-vs.-high design, showing a good segregation of the two subgroups. (**B**) Volcano plot based on RNA-Seq results of low-vs.-high design. (**C**) Top 20 DEGs in RNA-Seq in low-vs.-high design.

Pulmonary viral load was quantified by Nanostring with eight probes specific for SARS-CoV-2, and samples were divided into two groups, high and low viral load, numbering six and eight cases respectively, using the median value as a cutoff between the two groups (Figure 4). The two groups were moderately distinguished one from another, as regards DEGs identified by RNA-Seq. The PCA plot exemplifies the separation between these two with no significant difference between male and females.

The volcano plot in Figure 4B illustrates the DEGs obtained in low-vs-high design. It shows the names of specific genes with the highest degree of differential expression between the two groups. On the right side of the plot are the overexpressed genes in high viral load patients, while on the left side the underexpressed ones. Amongst the deregulated genes (Figure 4C), the significantly upregulated ones were *IFI44*, *IFI44L*, *IFI6*, *EPSTI1*, *CLEC4E*, *MX2*, *MX1*, *TLR2*, *IFIT2*, *FPR2*, *ZC3H12A*, *IFIT3*, *LOC10798*, *PARP9*, *DDX60L*, *ISG15*, *GBP2* and *GBP5*. On the other hand, differential expression analysis revealed a downregulation of *SFTPC* and *WWTR1*. Among these, *IFI44*, *IFI44L*, *IFI6*, *IFIT2*, *IFIT3*, *PARP9*, *DDX60L*, *ISG15*, *GBP2* and *GBP5* belong to INF I signaling pathway, while *EPSTI1*, *CLEC4E*, *MX2*, *MX1*, *TLR2*, *FPR2* and *ZC3H12A* can be associated with macrophage activation pathways; those not belonging to a specific category are *WWTR1* and *LOC10798*.

Despite not falling into a specific pathway category, *SFTPC* encodes the pulmonary-associated surfactant protein C (SP-C), essential for lung function, and is notably by far the most deregulated gene in terms of statistical significance (*q*-value = 6.41 × 10^−6^) and log Fold Change score (log2FC = 6.21).

### 3.6. Immunohistochemical Evaluation

**Figure 5:**
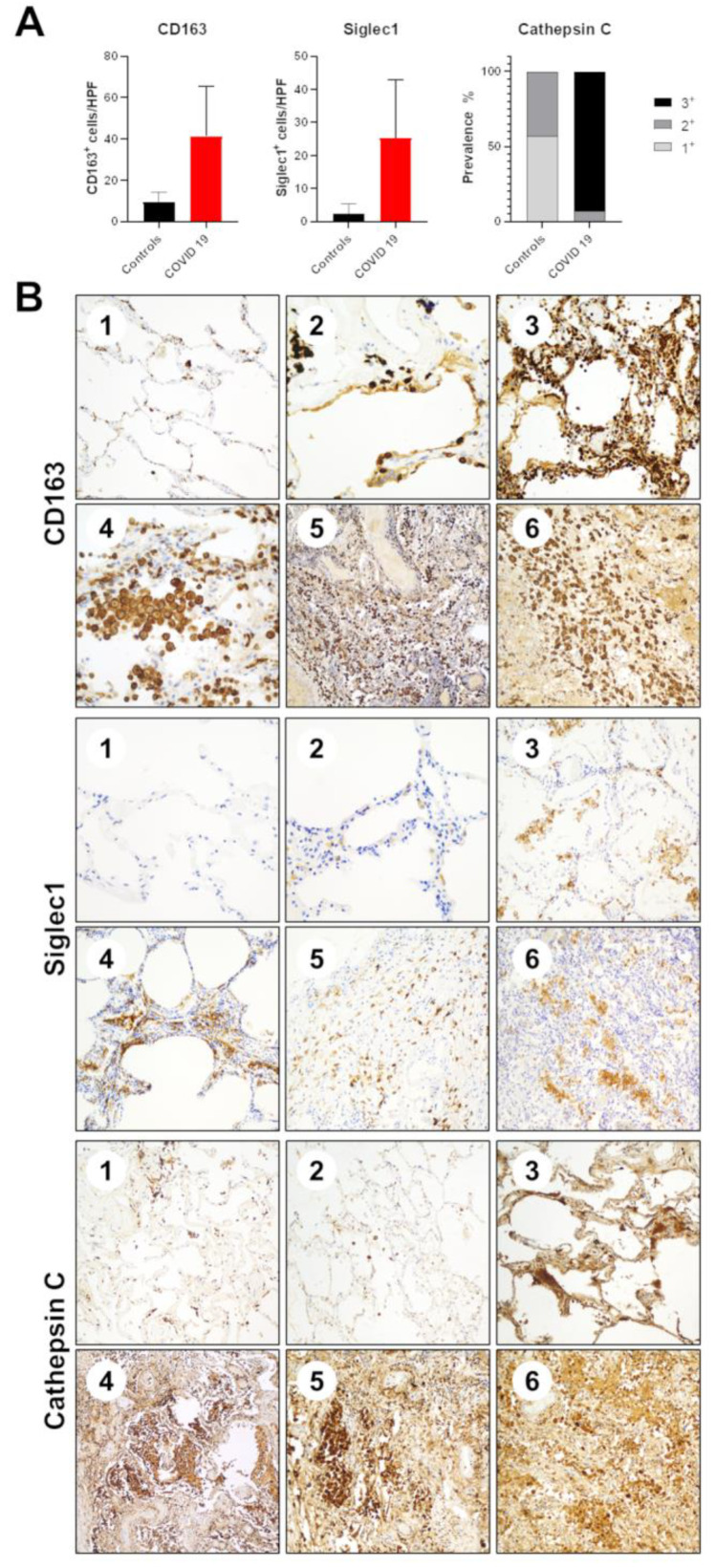
Immunohistochemical validation of gene expression profiling data. (**A**) Boxplots of the expression of CD163, Siglec-1 and Cathepsin C by means of IHC in samples and controls. (**B**) Representative IHC staining of CD163, Siglec-1 and Cathepsin C in control (1 and 2) and COVID-19 derived (3–6) samples.

Among the most deregulated genes, we performed IHC staining for CD163, Siglec-1 and Cathepsin-C (Figure 5) on the same 21 samples tested by gene expression profiling. All the three tested proteins confirmed the data obtained by Nanostring/total RNA-Seq profiling.

As for CD163, the mean of controls was 9.6 positive cells/HPF, while the mean of COVID-19 patients was 41.4 (*p* = 0.0029). As for Siglec-1, the mean of controls was 2.4 positive cells/HPF, while the mean of COVID-19 patients was 25.2 (*p* = 0.0035).

For cathepsin C IHC results, the median of controls was 1+, while the median of COVID-19 patients was 3+ (*p* < 0.0001).

## 4. Discussion

In this study we have analyzed the differential expression of a large number of immune and inflammatory-related transcripts in autopsy lung specimens of COVID-19 deceased patients, with the aim of identifying an immune signature associated with severe COVID-19. Our data confirm that severe COVID-19 is associated with hyperinflammation and a dysregulation of the innate and adaptive immune response. Specifically, SARS-CoV-2 specimens revealed upregulation of interferon-stimulated genes, monocytes/macrophage activation-associated genes, complement pathway and Cathepsin C.

Type I Interferon (IFN) is a key component to the immediate antiviral response. The IFN system comprises several IFN cytokines inducing hundreds of IFN stimulated effectors genes (ISG) [12]. It is well-established that severe COVID-19 is associated with an impaired IFN-I response. However, while low levels of IFNs have been detected in the peripheral blood of patients with severe COVID-19 [13], the local induction of IFNs and ISGs has been reported in the bronchoalveolar lavage (BAL) of some critically ill patients [14]. This discrepancy has been attributed to the activation of specialized immune cells such as lung-resident dendritic cells (DCs), which produce IFNα in response to SARS-CoV-2 infection [14]. Recent clinical trials have shown that early administration of IFN-α is linked to reduced mortality in hospitalized patients, while late IFN-α therapy leads to increased mortality and delayed recovery. Conceivably, high levels of IFN in severe COVID-19 might have no antiviral effect but promote inflammation and tissue damage [15].

Among the antiviral proteins induced by IFN1, the *OAS* genes harbor genetic variants that might influence susceptibility to the SARS-CoV infection and progression [16]. These genes are responsible for the production of a host antiviral mediator (2′,5′-oligoadenylate [2-5A]), that activates the effector enzyme RNAse L. RNAse L degrades a double-stranded RNA generated by the virus as a replication intermediate [17,18]. The OAS genes are also a potential therapeutic target: 2-5A is degraded by endogenous phosphodiesterase 12 (PDE-12). Therapeutic PDE-12 inhibitors are available and increase OAS-mediated antiviral activity [19].

Among the interferon-stimulated genes that were found to be upregulated in our analysis, the gene *ISG15* is of particular interest. The ISGylation is a host defense mechanism which involves the post-translational attachment of ubiquitin-like protein ISG15 to host and viral target substrates. SARS-CoV-2 triggers deISGylation to generate free ISG15 through the papain-like protease (PLpro) enzyme [20,21]. PLpro is an attractive drug target because it has multiple essential functions involved in processing viral proteins, with several compounds having shown promising results as an antiviral therapy in vitro [22,23].

Excessive monocyte/macrophage activation is another pillar of SARS-CoV-2 immune response and contributes to hyperinflammation [24]. Single-cell RNA sequencing analysis of BAL collected from patients with severe or mild COVID-19 revealed a marked expansion of the mononuclear phagocytes compartment. According to our results, mononuclear phagocyte composition was characterized by a depletion of tissue-resident alveolar macrophages and an enrichment of inflammatory monocyte-derived macrophages in critical patients [25]. Recent studies found low CD169/Siglec1 and high CD163 expression on circulating monocytes in severe COVID-19 patients [26,27]. The early phases of COVID-19 were characterized by an enrichment of CD169/Siglec1+ monocytes in the peripheral blood [26]. Conversely, our data show how the lung immune microenvironment of deceased COVID-19 patients is characterized by a range of macrophage activation states. In fact, the local enrichment of CD163+ monocytes is associated with anti-inflammatory macrophage functions [28], while the abundance CD169/Siglec1+ monocytes reflects IFN1 pathway activation [29].

Complement has emerged as a key contributor to the pathogenesis of COVID-19–associated tissue inflammation and thrombosis and is becoming an attractive therapeutic target [30]. In line with our results, there are several lines of evidence for local deposition of complement proteins and activation products in post mortem tissue samples showing activation of the three pathways (classical, alternative and mannose-binding lectin pathway) [31,32]. The lectin and alternative complement pathways are thought to be associated with COVID-19 mortality. However, the involvement of the complement cascade in COVID-19 seems to be heterogeneous and still to be fully elucidated [33].

Neutrophils play a crucial role in SARS-CoV-2 mediated lung damage by releasing elastase-related serine proteases and reactive oxygen [34]. Interestingly, our analysis found the gene *CTSC* to be upregulated in lung tissue of deceased COVID-19 patients. CTSC encodes for the cysteine dipeptidyl aminopeptidase Cathepsin C (Cat C), that activates most of tissue-degrading elastase-related serine proteases. For this reason, CatC is an attractive therapeutic target to prevent the irreversible pulmonary failure caused in patients with severe COVID-19 [35].

Differential expression analysis revealed lower levels in samples with higher viral load of *SFTPC*. Through the infection of type II alveolar cells, SARS-CoV-2 interferes with the production of pulmonary surfactant, of which surfactant protein C is a key component, thus causing an increase in surface tension, lastly driving to alveolar collapse [36]. In accordance with this, our data highlight a higher *SFTPC* expression in patients with lower viral load. As a direct consequence of this, the use of pulmonary surfactant as an additional therapy for the treatment of ARDS can be supported [36]. Moreover, surfactant protein C plays a role in regulating inflammation via JAK/STAT pathway and this may represent an additional therapeutic benefit [37].

The main limitations of the study are the relatively small sample size and the poor quality of the RNA due to the intrinsic poor preservation of autopsy tissue specimens. COVID-19 patients derived from the first pandemic wave in which autopsy was performed only in small series of patients with limited access and heterogeneous information on the clinical management of the disease and laboratory testing. Moreover, viral pathogenicity has changed over time and what we observed in this series should be validated in more recent cases of COVID-19-related deaths. Furthermore, the evaluation of additional control cohorts bearing forms of infectious and noninfectious interstitial pneumonitis may help to better define which molecular alterations are specific to SARS-CoV-2 infection. Notwithstanding these limitations, the validation of the nCounter analysis by total RNA-Seq, which quantified the expression of more than 30,000 transcripts, represents a strength of our results. Another important point is that the subanalysis according to viral load highlighted the results obtained in the comparison between COVID-19 and control samples, which further validate the finding that the infection was responsible for INF I signaling pathway overexpression and macrophages activation.

In summary, detailed transcriptomic analysis of autopsy lung tissue specimens reveals the presence of a strong proinflammatory signature. Our data highlight the key role of IFN1 as the main driver of the dysregulated inflammatory and immune microenvironment observed in the lungs of COVID-19. The current work is a preliminary study which provides the foundation to evaluate a larger series of autopsies, to better understand the immunopathology of severe COVID-19 and to identify novel therapeutic targets and biomarkers to optimize patient selection and the timing of treatment administration.

## Figures and Tables

**Table 1:** Clinico-pathological features of the considered series.

COVID-19 Cohort (17 Patients)	
Age (years)	82.8 ± 8.5 (69–97)
Sex	M 9:F 8
Hospitalization (days)	7.5 ± 6.7 (0–26)
Comorbidities:	
Hypertension	10/17 (58.8%)
Cardiovascular disease	6/17 (35.3%)
Obesity	2/17 (11.8%)
Diabetes	3/17 (17.6%)
**Control Cohort (7 Patients)**	
Age (years)	50.0 ± 4.7 (41–56)
Comorbidities:	
Hypertension	1/7 (14.3%)
Cardiovascular disease	7/7 (100%)
Obesity	0/7 (0%)
Diabetes	2/7 (38.6%)
Sex	M 7:F 0

**Table 2:** Detailed clinical and laboratory data of COVID-19 patients.

ID	Age (Years)	Gender (M/F)	Smoking	Comorbidities	Enoxaparin (Anticoagulant Therapy)	Antibiotic Therapy	Antiviral Therapy	Tolicizumab	Tracheal Intubation	Length of Invasive Ventilation (Days)	ICU	ICU Stay (Days)	Hospitalization (Days)	Lymphocyte Count (10^9^/L)	D-Dimer (ng/mL)	Fibrinogen (mg/dL)	IL-6 (ng/L)	Ferritin (ng/mL)	LDH (U/L)
1	82	M	No	Yes	Yes	Yes	Yes	N	N	-	Yes	1	2	0.51	311	na	na	na	427
2	69	F	No	Yes	Yes	Yes	Yes	No	Yes	5	Yes	5	4	1.14	55.744	0.8	na	na	406
3	76	M	No	Yes	No	Yes	Yes	No	Yes	5	Yes	5	4	1.3	315	6.8	66.8	485	369
4	71	M	No	Yes	Yes	Yes	No	No	Yes	1	Yes	1	5	9.88	684	3.3	na	na	na
5	87	F	Former	Yes	Yes	Yes	No	No	No	-	No	-	4	na	na	na	na	na	660
6	79	M	Yes	Yes	Yes	Yes	No	No	Yes	6	Yes	7	7	0.7	1276	7.6	210	4253	446
7	76	M	No	Yes	Yes	Yes	Yes	No	Yes	19	Yes	19	26	1.32	268	4.6	na	na	na
8	96	M	No	Yes	Yes	Yes	No	No	No	-	No	-	4	0.92	1189	7.6	299	2416	217
9	86	M	No	Yes	Yes	Yes	No	No	No	-	No	-	6	0.28	5329	na	na	915	434
10	90	F	No	Yes	Yes	Yes	No	No	No	-	No	-	4	1.09	4029	na	na	na	na
11	75	M	No	No	Yes	Yes	Yes	No	Yes	-	Yes	20	21	0.93	910	7.5	878	3089	104
12	88	F	No	Yes	Yes	Yes	No	No	No	-	No	-	6	0.95	222	na	na	524	219
13	74	F	Former	Yes	Yes	Yes	Yes	No	Yes	8	Yes	11	13	0.57	969	8.19	18	363	247
14	87	F	No	Yes	No	Yes	No	No	No	-	No	-	0	0.43	na	na	na	na	766
15	92	F	No	Yes	Yes	Yes	No	No	No	-	No	-	5	na	na	na	na	na	556
16	83	M	Former	Yes	Yes	Yes	Yes	No	No	-	No	-	8	0.35	na	na	na	na	na
17	97	F	No	Yes	Yes	Yes	No	No	No	-	No	-	9	0.52	507	4.1	na	669	286

**Table 3:** Common deregulated genes from nCounter and total RNA-Seq analyses.

#	Gene ID	Gene Name	Main Function	*p*-Value
**Common upregulated genes**				
1	*PKM*	Pyruvate Kinase M1/2	Catalyzes the last step within glycolysis, the dephosphorylation of phosphoenolpyruvate to pyruvate.	*p* < 0.01
2	*IFIT1*	Interferon Induced Protein with Tetratricopeptide Repeats 1	Acts as a sensor of viral single-stranded RNAs and inhibits expression of viral messenger RNAs.	*p* < 0.01
3	*OAS1*	2′-5′-Oligoadenylate Synthetase 1	Activates latent RNase L, which results in viral RNA degradation and the inhibition of viral replication.	*p* < 0.01
4	*C2*	Complement C2	Functions as part of the classical pathway of the complement system.	*p* < 0.01
5	*CFP*	Complement Factor B	Functions as part of the alternate pathway of the complement system.	*p* < 0.01
6	*SIGLEC1*	Sialic Acid Binding Ig Like Lectin 1	Mediates sialic-acid dependent binding of macrophages to lymphocytes, granulocytes, monocytes, natural killer cells, B-cells and CD8 T-cells.	*p* < 0.01
7	*CD163*	Cluster of Differentiation 163	Is involved in clearance and endocytosis of hemoglobin/haptoglobin complexes by macrophages. Acts as an innate immune sensor for bacteria.	*p* < 0.01
8	*ISG15*	Interferon-stimulated gene 15	Plays a key role in the innate immune response to viral infection either via its conjugation to a target protein (ISGylation) or via its action as a free or unconjugated protein.	*p* < 0.01
9	*CTSC*	Cathepsin C	Functions as a central coordinator for activation of many serine proteases in immune/inflammatory cells.	*p* < 0.01
10	*RSAD2*	Radical S-Adenosyl Methionine Domain Containing 2	Plays a major role in the cell antiviral state induced by type I and type II interferon	*p* < 0.01
11	*MS4A4A*	Membrane Spanning 4-Domains A4A	May be involved in signal transduction as a component of a multimeric receptor complex.	*p* < 0.01
**Common downregulated genes**				
12	*TCF4*	Transcription Factor 4	Acts as a transcription factor which binds to the immunoglobulin enhancer mu-E5/kappa-E2 motif.	*p* < 0.01
13	*SNCA*	Synuclein Alpha	Involved in synaptic vesicle recycling.	*p* < 0.01
14	*TGFB3*	Transforming Growth Factor Beta 3	A cytokine involved in cell differentiation, embryogenesis and development.	*p* < 0.01
15	*CD83*	Cluster of Differentiation 83	May play a significant role in antigen presentation.	*p* < 0.01
16	*DLL4*	Delta Like Canonical Notch Ligand 4	Encodes Notch ligands.	*p* < 0.01

## Data Availability

The data presented in our study are available on request from the corresponding author. The data are not publicly available due to our internal policy in terms of privacy restrictions.

## Supplementary Materials

The following supporting information can be downloaded at: https://www.mdpi.com/article/10.3390/cells11061011/s1, Figure S1: Phylogeny by nextclade: (A) SARS-CoV-2 phylogeny as described until 1 February 2022. (B) The detected clades in the six high viral load samples.

## References

[1] Tay M.Z., Poh C.M., Rénia L., Macary P.A., Ng L.F.P. (2020). The trinity of COVID-19: Immunity, inflammation and intervention. Nat. Rev. Immunol..

[2] Grasselli G., Tonetti T., Filippini C., Slutsky A.S., Pesenti A., Ranieri V.M. (2021). Pathophysiology of COVID-19-associated acute respiratory distress syndrome—Authors’ reply. Lancet Respir. Med..

[3] Huang C., Wang Y., Li X., Ren L., Zhao J., Hu Y., Zhang L., Fan G., Xu J., Gu X. (2020). Clinical features of patients infected with 2019 novel coronavirus in Wuhan, China. Lancet.

[4] Xu Z., Shi L., Wang Y., Zhang J., Huang L., Zhang C., Liu S., Zhao P., Liu H., Zhu L. (2020). Pathological findings of COVID-19 associated with acute respiratory distress syndrome. Lancet Respir. Med..

[5] Calabrese F., Pezzuto F., Fortarezza F., Hofman P., Kern I., Panizo A., von der Thüsen J., Timofeev S., Gorkiewicz G., Lunardi F. (2020). Pulmonary pathology and COVID-19: Lessons from autopsy. The experience of European Pulmonary Pathologists. Virchows Arch..

[6] Zarrilli G., Angerilli V., Businello G., Sbaraglia M., Traverso G., Fortarezza F., Rizzo S., De Gaspari M., Basso C., Calabrese F. (2021). The Immunopathological and Histological Landscape of COVID-19-Mediated Lung Injury. Int. J. Mol. Sci..

[7] Basso C., Calabrese F., Sbaraglia M., Del Vecchio C., Carretta G., Saieva A., Donato D., Flor L., Crisanti A., Tos A.P.D. (2020). Feasibility of postmortem examination in the era of COVID-19 pandemic: The experience of a Northeast Italy University Hospital. Virchows Arch..

[8] Basso C., Leone O., Rizzo S., De Gaspari M., van der Wal A.C., Aubry M.-C., Bois M.C., Lin P.T., Maleszewski J.J., Stone J.R. (2020). Pathological features of COVID-19-associated myocardial injury: A multicentre cardiovascular pathology study. Eur. Heart J..

[9] Calabrese F., Pezzuto F., Fortarezza F., Boscolo A., Lunardi F., Giraudo C., Cattelan A., Del Vecchio C., Lorenzoni G., Vedovelli L. (2021). Machine learning-based analysis of alveolar and vascular injury in SARS-CoV-2 acute respiratory failure. J. Pathol..

[10] Borczuk A.C., Salvatore S.P., Seshan S.V., Patel S.S., Bussel J.B., Mostyka M., Elsoukkary S., He B., Del Vecchio C., Fortarezza F. (2020). COVID-19 pulmonary pathology: A multi-institutional autopsy cohort from Italy and New York City. Mod. Pathol..

[11] Vukmirovic M., Herazo-Maya J.D., Blackmon J., Skodric-Trifunovic V., Jovanovic D., Pavlovic S., Stojsic J., Zeljkovic V., Yan X., Homer R. (2017). Identification and validation of differentially expressed transcripts by RNA-sequencing of formalin-fixed, paraffin-embedded (FFPE) lung tissue from patients with Idiopathic Pulmonary Fibrosis. BMC Pulm. Med..

[12] Barrat F.J., Crow M.K., Ivashkiv L.B. (2019). Interferon target-gene expression and epigenomic signatures in health and disease. Nat. Immunol..

[13] Hadjadj J., Yatim N., Barnabei L., Corneau A., Boussier J., Smith N., Péré H., Charbit B., Bondet V., Chenevier-Gobeaux C. (2020). Impaired type I interferon activity and inflammatory responses in severe COVID-19 patients. Science.

[14] Zhou Z., Ren L., Zhang L., Zhong J., Xiao Y., Jia Z., Guo L., Yang J., Wang C., Jiang S. (2020). Heightened Innate Immune Responses in the Respiratory Tract of COVID-19 Patients. Cell Host Microbe.

[15] Sodeifian F., Nikfarjam M., Kian N., Mohamed K., Rezaei N. (2021). The role of type I interferon in the treatment of COVID-19. J. Med. Virol..

[16] Pairo-Castineira E., Clohisey S., Klaric L., Bretherick A.D., Rawlik K., Pasko D., Walker S., Parkinson N., Fourman M.H., Russell C.D. (2021). Genetic mechanisms of critical illness in COVID-19. Nature.

[17] Hamano E., Hijikata M., Itoyama S., Quy T., Phi N.C., Long H.T., Ha L.D., Ban V.V., Matsushita I., Yanai H. (2005). Polymorphisms of interferon-inducible genes OAS-1 and MxA associated with SARS in the Vietnamese population. Biochem. Biophys. Res. Commun..

[18] He J., Feng D., De Vlas S.J., Wang H., Fontanet A., Zhang P., Plancoulaine S., Tang F., Zhan L., Yang H. (2006). Association of SARS susceptibility with single nucleic acid polymorphisms of OAS1 and MxA genes: A case-control study. BMC Infect. Dis..

[19] Wood E.R., Bledsoe R., Chai J., Daka P., Deng H., Ding Y., Harris-Gurley S., Kryn L.H., Nartey E., Nichols J. (2015). The Role of Phosphodiesterase 12 (PDE12) as a Negative Regulator of the Innate Immune Response and the Discovery of Antiviral Inhibitors. J. Biol. Chem..

[20] Liu G., Lee J.H., Parker Z.M., Acharya D., Chiang J.J., van Gent M., Riedl W., Davis-Gardner M.E., Wies E., Chiang C. (2021). ISG15-dependent activation of the sensor MDA5 is antagonized by the SARS-CoV-2 papain-like protease to evade host innate immunity. Nat. Microbiol..

[21] Mathieu N., Paparisto E., Barr S., Spratt D. (2021). HERC5 and the ISGylation Pathway: Critical Modulators of the Antiviral Immune Response. Viruses.

[22] Clemente V., D’Arcy P., Bazzaro M. (2020). Deubiquitinating Enzymes in Coronaviruses and Possible Therapeutic Opportunities for COVID-19. Int. J. Mol. Sci..

[23] Shin D., Mukherjee R., Grewe D., Bojkova D., Baek K., Bhattacharya A., Schulz L., Widera M., Mehdipour A.R., Tascher G. (2020). Papain-like protease regulates SARS-CoV-2 viral spread and innate immunity. Nature.

[24] Shaath H., Vishnubalaji R., Elkord E., Alajez N.M. (2020). Single-Cell Transcriptome Analysis Highlights a Role for Neutrophils and Inflammatory Macrophages in the Pathogenesis of Severe COVID-19. Cells.

[25] Liao M., Liu Y., Yuan J., Wen Y., Xu G., Zhao J., Cheng L., Li J., Wang X., Wang F. (2020). Single-cell landscape of bronchoalveolar immune cells in patients with COVID-19. Nat. Med..

[26] Doehn J.M., Tabeling C., Biesen R., Saccomanno J., Madlung E., Pappe E., Gabriel F., Kurth F., Meisel C., Corman V.M. (2021). CD169/SIGLEC1 is expressed on circulating monocytes in COVID-19 and expression levels are associated with disease severity. Infection.

[27] Schulte-Schrepping J., Reusch N., Paclik D., Baßler K., Schlickeiser S., Zhang B., Krämer B., Krammer T., Brumhard S., Bonaguro L. (2020). Severe COVID-19 Is Marked by a Dysregulated Myeloid Cell Compartment. Cell.

[28] Fischer-Riepe L., Daber N., Schulte-Schrepping J., de Carvalho B.C., Russo A., Pohlen M., Fischer J., Chasan A.I., Wolf M., Ulas T. (2020). CD163 expression defines specific, IRF8-dependent, immune-modulatory macrophages in the bone marrow. J. Allergy Clin. Immunol..

[29] Bourgoin P., Biéchelé G., Belkacem I.A., Morange P., Malergue F. (2020). Role of the interferons in CD64 and CD169 expressions in whole blood: Relevance in the balance between viral- or bacterial-oriented immune responses. Immun. Inflamm. Dis..

[30] Java A., Apicelli A.J., Liszewski M.K., Coler-Reilly A., Atkinson J.P., Kim A.H., Kulkarni H.S. (2020). The complement system in COVID-19: Friend and foe?. JCI Insight.

[31] Macor P., Durigutto P., Mangogna A., Bussani R., De Maso L., D’Errico S., Zanon M., Pozzi N., Meroni P.L., Tedesco F. (2021). Multiple-Organ Complement Deposition on Vascular Endothelium in COVID-19 Patients. Biomedicines.

[32] Magro C., Mulvey J.J., Berlin D., Nuovo G., Salvatore S., Harp J., Baxter-Stoltzfus A., Laurence J. (2020). Complement associated microvascular injury and thrombosis in the pathogenesis of severe COVID-19 infection: A report of five cases. Transl. Res..

[33] Defendi F., Leroy C., Epaulard O., Clavarino G., Vilotitch A., Le Marechal M., Jacob M.-C., Raskovalova T., Pernollet M., Le Gouellec A. (2021). Complement Alternative and Mannose-Binding Lectin Pathway Activation Is Associated with COVID-19 Mortality. Front. Immunol..

[34] Korkmaz B., Caughey G.H., Chapple I., Gauthier F., Hirschfeld J., Jenne D.E., Kettritz R., Lalmanach G., Lamort A.-S., Lauritzen C. (2018). Therapeutic targeting of cathepsin C: From pathophysiology to treatment. Pharmacol. Ther..

[35] Korkmaz B., Lesner A., Marchand-Adam S., Moss C., Jenne D.E. (2020). Lung Protection by Cathepsin C Inhibition: A New Hope for COVID-19 and ARDS?. J. Med. Chem..

[36] Cattel F., Giordano S., Bertiond C., Lupia T., Corcione S., Scaldaferri M., Angelone L., De Rosa F.G. (2021). Use of exogenous pulmonary surfactant in acute respiratory distress syndrome (ARDS): Role in SARS-CoV-2-related lung injury. Respir. Physiol. Neurobiol..

[37] Jin H., Ciechanowicz A., Kaplan A., Wang L., Zhang P.-X., Lu Y.-C., Tobin R.E., Tobin B.A., Cohn L., Zeiss C.J. (2018). Surfactant protein C dampens inflammation by decreasing JAK/STAT activation during lung repair. Am. J. Physiol. Cell. Mol. Physiol..

