# Supplementary material for: Multi-Design Differential Expression Profiling of COVID-19 Lung Autopsy Specimens Reveals Significantly Deregulated Inflammatory Pathways and SFTPC Impaired Transcription
Source: Cells. 2022 Mar 16;11(6):1011. doi: 10.3390/cells11061011 (PMC8947344; doi:10.3390/cells11061011)
Supplement: Supplementary file 1 [file cells-11-01011-s001.zip › cells-1631535-supplementary.pdf]

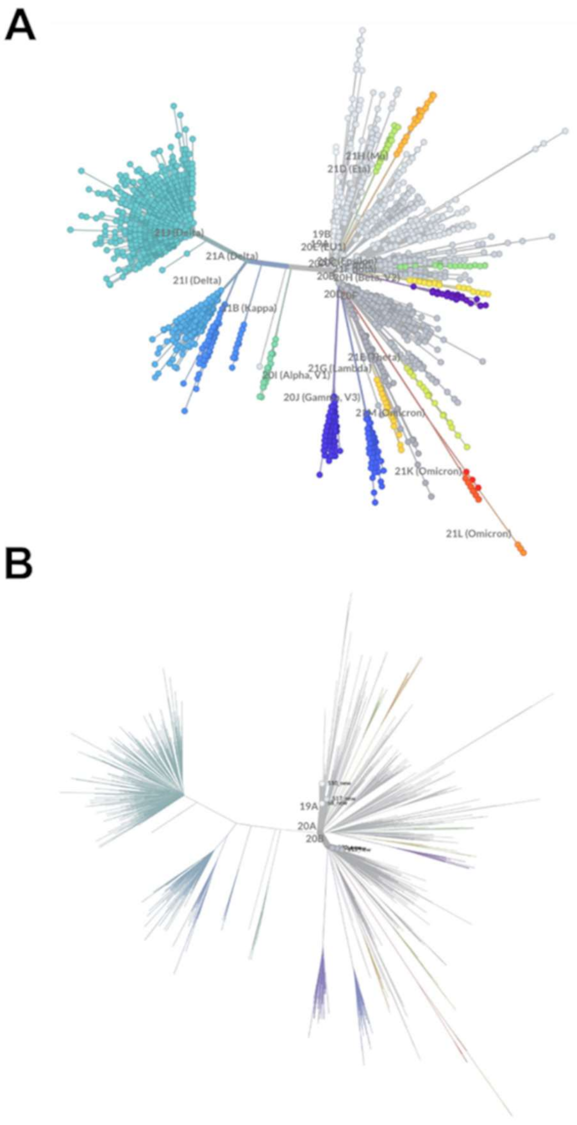

Figure S1: Phylogeny by nextclade: **(A)** SARS-CoV-2 phylogeny as described until 1st February 2022. **(B)** The detected clades in the six high viral load samples.
